# Effect of Prior Moderate Aerobic Exercise to Prolonged Sitting on Peripheral and Central Cardiovascular Measures in Young Women

**DOI:** 10.3390/jcdd11100307

**Published:** 2024-10-03

**Authors:** Abdullah Bandar Alansare, Rawan Tuayes Alotaibi, Ali Mufrih Albarrati, Lee Stoner, Bethany Barone Gibbs

**Affiliations:** 1 Department of Exercise Physiology, College of Sport Sciences and Physical Activity, King Saud University, King Khalid Rd., Riyadh 80200, Saudi Arabia; ronzaotb3@gmail.com; 2 Rehabilitation Sciences Department, College of Applied Medical Sciences, King Saud University, King Khalid Rd., Riyadh 80200, Saudi Arabia; albarrati@ksu.edu.sa; 3 Department of Sport and Exercise, University of North Carolina, Chapel Hill, NC 27599, USA; stonerl@email.unc.edu; 4 Department of Epidemiology and Biostatistics, West Virginia University School of Public Health, Morgantown, WV 26506, USA; bethany.gibbs@hsc.wvu.edu

**Keywords:** blood pressure, pulse wave velocity, heart rate variability, sedentary lifestyle, physical activity

## Abstract

Background: Prolonged sitting is a risk factor for cardiovascular disease (CVD). We examined whether moderate aerobic exercise prior to prolonged sitting (EX + SIT) has protective effects on peripheral and central cardiovascular and autonomic measures. Methods: Young women (*n* = 26; 23.4 ± 4.3 years old; BMI = 23.1 ± 4.3) completed two sessions in random order: (1) EX + SIT, which consisted of 25 min of moderate aerobic exercise followed by a 3 h prolonged sitting bout, and (2) a 3 h prolonged sitting bout only (SIT-only). Seated peripheral and central blood pressure (BP), pulse wave velocity (PWV), and heart rate variability (HRV) were measured at baseline and after 1 h, 2 h, and 3 h of sitting. Generalized linear mixed models with random effects examined the effects of conditions (i.e., EX + SIT vs. SIT) on BP, PWV, and HRV while adjusting for baseline values. Results: Only peripheral and central diastolic BP (β = 2.18; *p* = 0.016 and β = 1.99; *p* = 0.034, respectively) were significantly lower in the EX + SIT condition compared to the SIT-only condition. No differences were detected in other BP, PWV, or HRV variables between the two conditions (*p* > 0.05 for all). Conclusions: Performing moderate aerobic exercise in the morning before engaging in prolonged sitting bouts may reduce some of the prolonged-sitting-induced cardiovascular impairments in young women. Further research is needed to confirm these findings in males and middle-aged/older adults.

## 1. Introduction

Sedentary behavior (SB) such as prolonged, uninterrupted sitting is increasing at an alarming rate worldwide [1,2]. SB is now recognized as a significant risk factor for cardiovascular diseases (CVDs) and mortality [3]. Strong evidence suggests that the associations of SB with CVDs and mortality may be explained by several unfavorable cardiovascular and autonomic responses to prolonged sitting [3,4,5]. For instance, acute bouts of prolonged, uninterrupted sitting increase CVD risk factors such as blood pressure (BP) and pulse wave velocity (PWV), a non-invasive measure of vascular stiffness [6,7,8,9]. Over time, persistent elevations of BP and PWV may lead to CVD and mortality. As such, public health efforts to mitigate the adverse effects of too much SB have emerged, including the construction of local and global recommendations to reduce SB and prolonged SB patterns [10,11,12].

In general, adults are recommended to limit overall SB and take frequent breaks during prolonged sitting bouts [10,11,12]. Strategies to break up prolonged sitting include intermittent standing [13], short bouts of walking or other aerobic exercise [13,14], or bouts of resistance exercise [15]. However, in some work scenarios (e.g., official meetings) and often while traveling or commuting, these strategies are not feasible, and individuals may be required to engage in prolonged sitting and the associated health hazards. As such, exploring alternative approaches to combat the negative impacts of prolonged sitting is necessary to improve health and prevent cardiovascular hazards.

Some research has explored whether exercise prior to prolonged sitting is an effective strategy to prevent the unfavorable cardiovascular alterations that are induced by prolonged sitting. However, the effects of exercise prior to prolonged sitting on CVD risk factors are inconsistent. For example, recent investigations revealed that prolonged-sitting-induced endothelial dysfunctions were prevented by performing aerobic exercise prior to prolonged sitting in young adults [16,17]. Nonetheless, implementing aerobic exercise prior to prolonged sitting did not attenuate BP or PWV increases in young males or mixed-sex samples [18,19,20]. Yet, importantly, previous research has indicated that aerobic exercise breaks (i.e., those performed during prolonged sitting bouts) attenuate BP responses to prolonged sitting to a greater extent in women than in men [14]. Accordingly, the inclusion of only males or mixed samples could have masked the beneficial cardiovascular effects of aerobic exercise prior to prolonged sitting on BP and PWV in previous studies [18,19]. As such, further research that examines the effects of exercise prior to prolonged sitting based on various cardiovascular and autonomic measurements taken from female samples is needed to achieve a more comprehensive understanding of its cardiovascular and autonomic benefits. Furthermore, including only young females is of particular importance, as recent data suggest that young-to-middle-aged females have experienced no improvements in rates of CVD, while similarly aged males and older females have experienced decreasing CVD rates [21,22].

Therefore, this study aims to examine whether moderate aerobic exercise prior to prolonged sitting (EX + SIT) attenuates the effects of prolonged sitting (SIT) on peripheral and central BP, PWV, and HRV in young women. It was hypothesized that performing moderate aerobic exercise prior to prolonged sitting would have a protective effect on cardiovascular and autonomic measures compared to prolonged sitting that did not occur after aerobic exercise.

## 2. Materials and Methods

This study was reported following CONSORT (Consolidated Standards of Reporting Trials) guidelines [23]. The ethical approval was obtained from the Institutional Review Board at King Saudi University (No. 23/0074/IRB-A). All participants provided written informed consent before participating in the study.

### 2.1. Participants

Potential participants were recruited via advertisements posted in King Saud University buildings, sent via emails, and posted on social media platforms. Participants responding to advertisements were enrolled in the study if they met the following inclusion criteria: women aged from 18 to 40 years old, with resting systolic BP (SBP) ≤ 139 mmHg and resting diastolic BP (DBP) ≤ 89 mmHg [24], with a body mass index (BMI) < 30 kg/m^2^, who self-reported being able to perform prolonged sitting and moderate aerobic exercise, were free of any disease that may affect the study’s outcomes, and were not using antihypertensive or glucose-lowering drugs. Participants were excluded during screening if they were currently pregnant or breastfeeding. The entire study took place in the Exercise Physiology Laboratory at King Saud University.

### 2.2. Experimental Design

**Figure 1:**
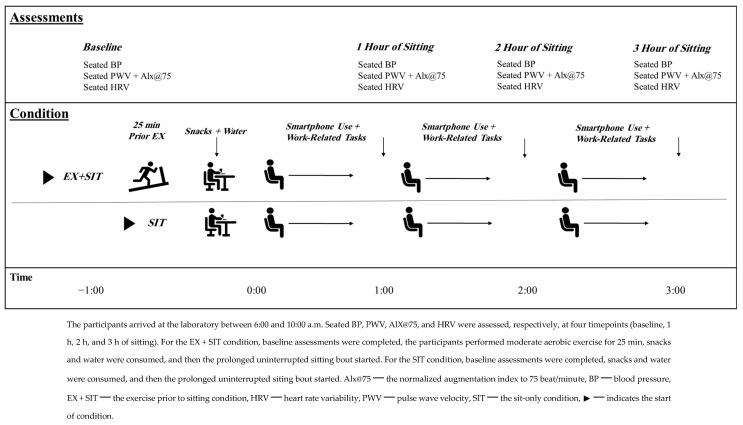
Experimental conditions.

This randomized cross-over study consisted of two experimental conditions that were performed on two different days and separated by a one-week interval [19,25] (Figure 1). Before each visit, the participants were instructed to refrain from consuming food for 12 h and to abstain from caffeine and self-perceived heavy exercise for 24 h. These abstentions were verbally confirmed upon arrival (between 6:00 am and 10:00 am). After informed consent was provided, baseline measurements, including BP eligibility measurements, were performed. Participants who met the study’s criteria were randomly assigned to perform a moderate aerobic exercise followed by 3 h of uninterrupted sitting (EX + SIT) or 3 h of uninterrupted sitting only (SIT). Cardiovascular measurements, including BP, PWV, and HRV, were obtained at four timepoints (i.e., baseline and after 1, 2, and 3 h of sitting). The same measurement procedures were followed during the two experimental visits for all participants.

### 2.3. Randomization

A simple randomization technique was performed to assign the order of experimental conditions for each participant. Research personnel wrote EX + SIT or SIT on two pieces of paper that were folded to hide the writing. The participant picked up one piece of paper and completed the selected condition.

### 2.4. Sample Size

The required sample size for the current study was calculated using the G Power software (G* Power Version 3.1.9.4), as follows. Assuming a within-subject correlation of 0.7, a type I error rate of 0.05, and 80% power, 21 participants were required to detect a standardized effect size of 0.25 within participants across the two experimental conditions (i.e., EX + SIT and SIT). To account for potential missing data or withdrawal, 28 participants were recruited.

### 2.5. Prolonged Sitting

During the study, each participant performed two separate bouts of prolonged, uninterrupted sitting. One bout was performed during the EX + SIT condition and the other was completed during the SIT condition. Each sitting bout lasted for three consecutive hours. Before starting, the participants were instructed to empty their bladders, if needed. Furthermore, during both sitting bouts, participants sat on a chair with the soles of both feet flat on the floor while maintaining a 90-degree angle at the knee and pelvic joints. Throughout the sitting period, the participants were allowed to use their smartphones and were provided with a computer to complete work-related tasks.

### 2.6. Exercise Session

During the EX + SIT condition, each participant performed aerobic exercise on a treadmill (h/p/cosmos, Inc., Nußdorf, Germany^®^) prior to the prolonged sitting bout. The exercise intensity was selected based on two factors: the participant’s safety and previous evidence suggesting that the chosen intensity can improve cardiovascular outcomes. To achieve this, the relative moderate intensity of 60% heart rate max (HR_max_) was selected [26,27,28]. The targeted heart rate was determined by using the following formula: 60%HR_max_ = (220 − age) * 0.6 [27]. According to the American College of Sports Medicine, this intensity is classified as light-intensity aerobic exercise [27]. Each participant performed moderate aerobic exercise for 25 min while following the Balke protocol [29]. In addition to its beneficial effects on health [30], this duration was selected because it is a manageable starting point for many young females, which promotes achieving the current physical activity recommendation (i.e., 150 min/week of moderate aerobic exercise throughout the week) [31].

To elaborate, the participants began with a 5 min walking warm-up. Next, the participants jogged or ran at a constant speed (5.3 km/h) for 25 min. The incline of the treadmill began at 0% and increased by 1% every minute, when needed, to ensure that each participant was exercising at their targeted intensity (i.e., 60%HR_max_) during the entire exercise period, and the participant’s heart rate was monitored with a chest heart rate strap (T31; Polar Electro, Inc., Helsinki, Finland^®^). Afterward, the participants performed a 5 min walk to cool down.

### 2.7. Snacks and Water

During the two experimental conditions, the participants were provided with a standardized snack and water (i.e., 16 ounces) to minimize potential distractions such as hunger, hypoglycemia, or physiological effects on the cardiovascular system while fasting. The snacks provided were Nature Valley^®^ (Minneapolis, MN, USA) oats and honey granola bars, which consisted of 190 calories per bar. Each participant received an individualized amount of the bars which fulfilled 30% of their total daily energy needs, which was determined by the Harris–Benedict equation [32]. The snack and water were provided to the participants during both experimental conditions, just before the start of the prolonged uninterrupted sitting bouts (Figure 1).

### 2.8. Measurements

#### 2.8.1. Participants’ Characteristics and Physical Activity Level

The participants self-reported their age and education level. Body height was measured in duplicate by a wall-mounted stadiometer (Perspective Enterprises, Portage, MI, USA), whereas body weight was measured in duplicate by a digital scale (WB-110A, Tanita, Tokyo, Japan). These duplicated measures were averaged to calculate the body mass index (BMI) (BMI = body weight in kg/body height in m^2^). The short version of the International Physical Activity Questionnaire was utilized to estimate the number of minutes participants spent performing MVPA.

#### 2.8.2. Peripheral Blood Pressure

Seated BP measurements were completed using a validated oscillometric device (MobilOGraph 24 h PWA Monitor^®^, Aachen, Germany) with an appropriately sized cuff based on the arm circumcenter [33,34]. The monitor was programmed to operate automatically. To begin the BP measurements, the participants sat in a chair for 10 min with their back supported, both feet on the floor, and both arms supported at their heart level. The cuff was placed on the left arm and two consecutive BP measurements were performed with a one-minute rest interval in between [34]. The average of the two measurements during the first assessment was used to determine eligibility. The same procedures were utilized to complete the BP measurements at baseline and each hour during the sitting protocol during both experimental conditions (Figure 1). After completing these measurements, the oscillometric device (MobilOGraph 24 h PWA Monitor^®^, Aachen, Germany) was connected to the IEM on Life’s Side software through Bluetooth to process and calculate the peripheral BP values, which included SBP, DBP, and mean arterial pressure (MAP).

#### 2.8.3. Central Blood Pressure, Pulse Wave Velocity, and Augmentation Index

The MobilOGraph 24 h PWA Monitor is also a valid oscillometric device that is commonly used to estimate the central BP, PWV, and augmentation index by using the cuff-based method [35]. After acquiring the seated peripheral BP measurements, the device inflates the cuff to the DBP value and records pulse waves for 10 consecutive seconds [36,37]. Then, an algorithm called ARCSolver (Austrian Institute of Technology, Vienna, Austria) estimates several variables, including seated central SBP (cSPB), DBP (cDBP), pulse pressure (cPP), PWV, augmentation pressure (AP), and the normalized augmentation index to 75 beat/min (Alx@75) [34,38]. In parallel with the peripheral BP measurements, these central cardiovascular estimations were performed at baseline and each hour during the sitting protocol during both experimental conditions (Figure 1). To be included in the current analysis, each participant was required to have at least one successful pulse wave analysis for each timepoint.

#### 2.8.4. Heart Rate Variability

The time intervals between consecutive heartbeats were measured to calculate seated HRV indices by using a validated instrument (Polar V800 monitor with Polar H10 heart rate strap; Polar Electro, Inc., Helsinki, Finland^®^) [39]. The heart rate strap was placed on the participants’ chest under their chest muscles while they were resting and prior to the BP measurements. During the measurement, participants were instructed to remain quiet, breathe normally, and not to move or to talk. A 5 min record of time intervals was collected at each timepoint (i.e., at baseline and every hour into sitting) during both experimental conditions (Figure 1).

To process and derive HRV indices, the time interval data were downloaded using PolarFlow. These data were imported into Kubios Premium analysis software (version 3.3.1, MATLAB, The MathWorks, Inc. Portola Valley, CA, United States of America). To clean the data, the current guidelines [40] were utilized as follows: (1) the automatic correction was used to ensure data had ≤5% artifacts, and (2) further visual inspection of the existence of any distortion in the data was performed. Then, two HRV indices were calculated, including the standard deviation of normal R-R intervals (SDNN) (i.e., a measure of overall variability) and the root mean square of successive differences (RMSSDs) (i.e., a measure of cardiac parasympathetic activity). Natural log transformation was performed for SDNN and RMSSD due to skewness. These HRV indices were chosen in the current study due to their ability to predict CVD, as well as its well-understood statistical and physiological basis [40].

### 2.9. Statistical Analysis

The characteristics of the participants were summarized as means and standard deviations (SDs) or frequencies and percentages (%), as appropriate. To assess the study’s hypotheses, generalized linear mixed (GLM) models with random effects examined the effects of conditions (i.e., EX + SIT vs. SIT) on BP, PWV, and HRV variables while adjusting for baseline values [41]. The estimated β coefficients in these models represented whether the average of the outcome was lower during the EX + SIT condition than the SIT condition. In addition, the strength of the effects was evaluated by calculating Cohen’s *d* as follows: *d* = β/the standard deviation of baseline values of the cardiovascular and autonomic variables. The magnitude of effects was considered large (*d* = 0.8), medium (*d* = 0.5), or small (*d* = 0.2) using conventional thresholds [42]. The statistical significance level was set as α < 0.05. Stata version 14 (StataCorp, LLC, College Station, TX, USA) was utilized for the data analyses.

## 3. Results

Out of the 28 women who were enrolled, 2 participants did not complete both experimental conditions (i.e., EX + SIT and SIT). Thus, a sample of 26 participants was analyzed and included in the presented results. These participants had complete data for HRV variables (*n* = 26), though only *n* = 25 participants were included in the BP and PWV analyses because one participant had no peripheral and central BP and PWV data for a condition (i.e., EX + SIT) due to a technical error. Participant characteristics are reported in Table 1. Overall, the participants tended to be young with healthy BMIs, BPs, and PWVs.

### Cardiovascular Responses to Prolonged Sitting with and without Prior Exercise

**Figure 2:**
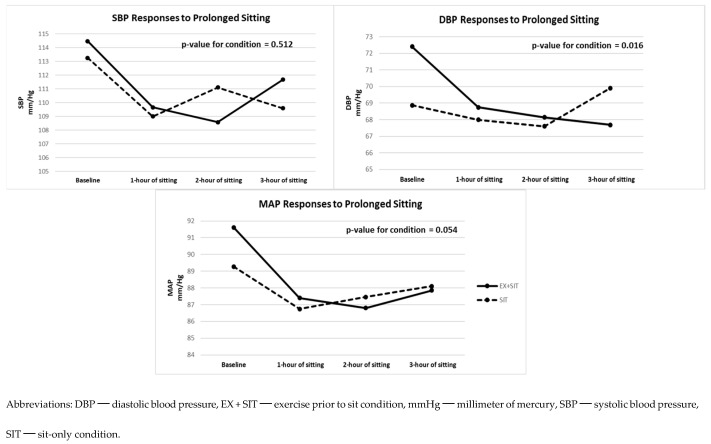
Peripheral BP responses to prolonged sitting with and without prior exercise (*n* = 25).

**Figure 3:**
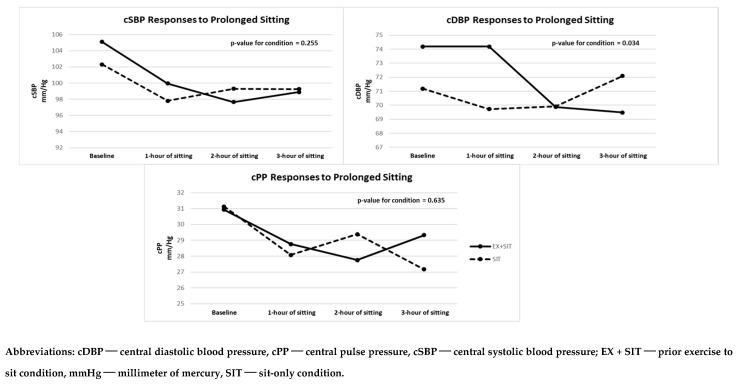
Central BP responses to prolonged sitting with and without prior exercise (*n* = 25).

Figure 2 and Supplemental Table S1 display peripheral BP differences between the EX + SIT vs. SIT condition. Both SBP (β = 0.77; *p* = 0.512) and MAP (β = 1.53; *p* = 0.054) were not significantly different between the two conditions. However, DBP (β = 2.18; *p* = 0.016) was significantly lower in the EX + SIT condition compared to the SIT condition. The size of this difference was small (*d* = 0.29). Furthermore, Figure 3 and Supplemental Table S1 show central BP differences between the two conditions. Neither cSBP (β = 1.39; *p* = 0.255) nor cPP (β = −0.48; *p* = 0.635) significantly differed when comparing the EX + SIT vs. SIT condition. Yet, cDBP (β = 1.99; *p* = 0.034) was significantly lower in the EX + SIT condition compared to the SIT condition. The size of this difference was mild (*d* = 0.26).

**Figure 4:**
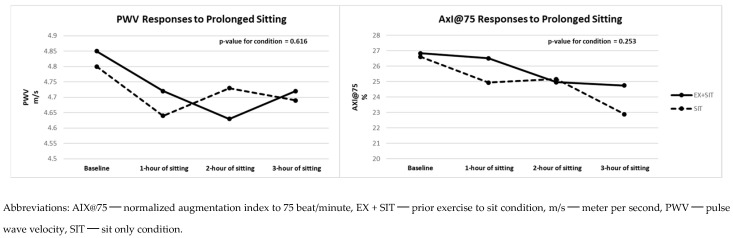
Vascular stiffness responses to prolonged sitting with and without prior exercise (*n* = 25).

**Figure 5:**
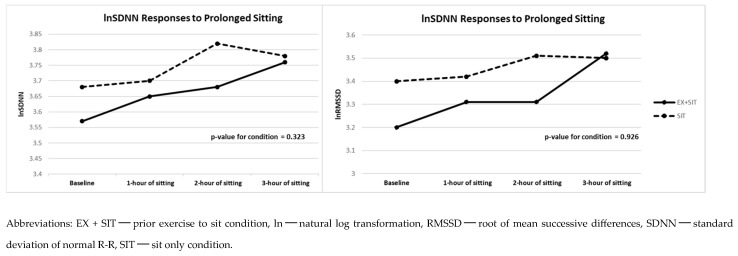
HRV responses to prolonged sitting with and without prior exercise (*n* = 26).

Figure 4 and Figure 5 and Supplemental Table S1 also reveal the vascular stiffness and HRV differences between the two conditions. No significant differences were observed in PWV (β = 0.02; *p* = 0.616), AIX@75 (β = −0.99; *p* = 0.253), lnSDNN (β = 0.04; *p* = 0.323), or lnRMSSD (β = 0.01; *p* = 0.926) between the EX + SIT vs. SIT condition.

## 4. Discussion

This study uniquely assessed seated peripheral and central cardiovascular and autonomic responses to EX + SIT vs. SIT-only conditions in young women. We revealed favorable effects of EX + SIT on peripheral and central DBP in young women; the sizes of these effects were small (*d* < 0.3 for both measures). Yet, other cardiovascular and autonomic measures were comparable when comparing the EX + SIT with the SIT-only condition. These findings suggest that while prolonged sitting may detrimentally affect cardiovascular and autonomic health, performing EX + SIT may be an effective strategy to prevent prolonged-sitting-induced DBP increases in young women.

### 4.1. Strengths and Limitations of the Study

This study has notable strengths. First, previous evidence suggested that there are sex differences in cardiovascular responses to aerobic exercise during prolonged sitting, with women having greater cardiovascular benefits [14]. Accordingly, this study included female participants only to clarify sex-specific effects. Moreover, the randomized cross-over (within-subject) design is another strength of the study, which allowed us to control for potential inter-participant variability [43]. In contrast to previous prolonged sitting studies [6,7,14], we simultaneously assessed peripheral and central cardiovascular and autonomic variables. Thus, our study provides a more comprehensive understanding of the cardiovascular and autonomic responses to prolonged sitting.

Nevertheless, a few drawbacks should be considered when interpreting the study’s findings. First, we did not measure or control for the menstrual cycle of the included participants. Although it is suggested not to control for the menstrual cycle in cardiovascular research to improve the external validity of the results [44], the counterpoint view suggests controlling for it to avoid the direct and indirect influence of ovarian hormones on cardiovascular regulations [45]. As such, future studies could account for such a debatable viewpoint. Moreover, our study enrolled healthy young women only. Thus, the effects of EX + SIT on peripheral and central cardiovascular and autonomic health may be different in older women, individuals with cardiovascular or other chronic diseases, or in their male counterparts [14,46]. Therefore, further investigations that enroll participants with these different characteristics are warranted.

### 4.2. Peripheral Cardiovascular Responses

To the best of our knowledge, only two existing studies have examined the effect of prior aerobic exercise on peripheral BP responses to prolonged sitting. The first study included a mixed-sex sample of young adults (*n* = 10; female *n* = 4) and reported that 30 min of moderate aerobic exercise significantly increased seated peripheral MAP during a 5 h prolonged, uninterrupted sitting bout compared to no prior exercise condition [20]. The other study enrolled young men only (*n* = 15) and found that 30 min of moderate aerobic exercise did not influence seated peripheral SBP, DBP, or MAP during prolonged sitting [18]. In contrast to these findings, we observed significantly lower seated peripheral DBP when 25 min of moderate aerobic exercise was performed prior to a 3 h prolonged sitting bout compared to no prior exercise condition in young women (*n* = 25).

The discrepancies observed between these studies may be most likely explained by sex and exercise modality differences. For example, the previous two studies enrolled a mixed-sex sample [20] or men only [18], whereas our study included women only. As mentioned earlier, data suggest the existence of sex differences in response to prolonged sitting [47] and aerobic exercise during prolonged sitting, with females likely acquiring greater cardiovascular benefits [14]. Moreover, while we observed significantly lower seated peripheral BP (i.e., DBP) when young women performed moderate aerobic exercise on a treadmill (i.e., jogging or running at 60% HRmax), the study that included a mixed-sex sample of young adults (i.e., men and women) found significantly higher seated peripheral BP (i.e., MPA) when moderate aerobic exercise was performed on a cycling ergometer (i.e., cycling at 90% power-evoking gas exchange threshold) [20]. Robust evidence indicates that the favorable effects of aerobic exercise on peripheral BP are much clearer and stronger when exercise is performed on a treadmill vs. a cycling ergometer [48,49,50]. Together, these findings suggest that the evidence about the effects of prior exercise to prolonged sitting on peripheral cardiovascular health remains mixed and warrants further investigation.

### 4.3. Central Cardiovascular and Autonomic Responses

Comparable to the prolonged sitting and peripheral cardiovascular health literature, no existing study examined the influence of aerobic exercise prior to prolonged sitting on central autonomic health and only two recent studies assessed the effects on central cardiovascular measures [18,19]. The first study examined the influence of 30 min of moderate aerobic exercise prior to a 3 h prolonged sitting bout on central seated SBP, DBP, MAP, and supine carotid–femoral PWV (cfPWV) in young men [18]. It was revealed that moderate aerobic exercise prevented prolonged-sitting-induced increases in central SBP, DBP, and MAP but failed to mitigate cfPWV increase [18]. Conversely, the other study found that 30 min of moderate aerobic exercise prior to a 2.5 h prolonged sitting bout prevented an increase in supine brachial–femoral PWV (bfPWV) in a sample of mixed young adults (*n* = 22; 50% males); yet it failed to prevent the supine femoral–ankle PWV (faPWV) increase [19]. In our current study, performing 25 min of moderate aerobic exercise prior to a 3 h prolonged sitting bout decreased central seated DBP in young women (*n* = 25). However, neither of the other central seated BP values, nor seated PWV or HRV, were significantly different from the SIT-only condition. In summary, the current evidence suggests that performing aerobic exercise prior to prolonged sitting may prevent prolonged-sitting-induced central DBP increases in male and female adults. However, the responses of SBP, central vascular stiffness, and autonomic measures to moderate aerobic exercise prior to prolonged sitting remain variable. Possible reasons for disparate results require further research and may include sex and measurement posture [6,51].

### 4.4. Potential Mechanisms

Previous studies showed that a single bout of prolonged sitting can reduce venous return, stroke volume, cardiac output, and vasoactive substances and increase sympathetic activation, leading to increased BP [7,51]. In contrast, a single bout of moderate aerobic exercise can potentially reverse these adverse cardiovascular changes, leading to reduced BP—a phenomenon known as post-exercise hypotension [52]. In our current study, all cardiovascular risk factors seem to oppose findings from previous studies [7,51] and showed improvements from baseline across the prolonged sitting bout, regardless of the prior exercise. Even though explanations for these discrepancies appear to be complex and need further investigation, a previous systematic review and meta-analysis suggest that the contradicting cardiovascular responses to movement behaviors, such as exercise in adults, may be explained by ethnicity and genes [53]. Nevertheless, peripheral and central DBPs were significantly lower in the EX + SIT vs. SIT-only condition. Although these effects do not appear to be the result of improved autonomic regulation (i.e., unchanged HRV), they may be due to reduced vascular resistance during the moderate aerobic exercise which persisted into the prolonged sitting bout. However, further research is needed to confirm this hypothesis.

### 4.5. Clinical Significance

The current SB recommendations suggest limiting SB but do not specify the best approaches to confront the adverse impacts of excessive, unavoidable SB on cardiovascular health [10,11,12]. Individuals are suggested to take frequent breaks during prolonged sitting bouts. While this recommendation may work in circumstances such as during leisure time, it may be challenging in other times such as during formal meetings and while driving. Our study provides preliminary evidence that performing moderate aerobic exercise in the morning before engaging in prolonged sitting bouts may be an alternative strategy to partially avert prolonged-sitting-induced BP increases, particularly for DBP in young women. These findings add further evidence and strategy to the current SB recommendations that aim at improving cardiovascular health.

## 5. Conclusions

In summary, this study uniquely assessed the effects of EX + SIT vs. SIT-only on peripheral and central cardiovascular and autonomic measures in young women. As hypothesized, EX + SIT appeared to have favorable effects on peripheral and central DBP. However, no effects on other measures of peripheral or central BP, PWV, or HRV were observed. As such, performing moderate aerobic exercise in the morning before engaging in prolonged sitting bouts may confront some but not all of the prolonged-sitting-induced cardiovascular impairments. Further research is needed to confirm these findings in different population groups, such as older women, and explore the potential mechanisms.

## Figures and Tables

**Table 1:** Participant characteristics (*n* = 26).

Variable	Mean (SD) or *n* (%)
Age, years	23.4 (4.3)
Education	
High School Degree or less	18 (69.2)
Bachelor’s Degree or higher	8 (30.8)
MVPA, min/week	82.5 (79.3)
Height, cm	158.7 (6.5)
Weight, kg	58.3 (11.9)
BMI, kg/m^2^	23.1 (4.3)
SBP, mmHg *	113.6 (9.7)
DBP, mmHg *	72.1 (7.5)
MAP, mmHg *	91.0 (8.0)
cSPB, mmHg *	103.7 (9.1)
cDBP, mmHg *	72.7 (7.8)
cPP, mmHg *	31.0 (6.2)
PWV, m/s *	4.8 (0.4)
AIX@75, % *	26.7 (8.8)
SDNN, ln	3.6 (0.3)
RMSSD, ln	3.3 (0.5)

Abbreviations: AIX@75—normalized augmentation index to 75 beat/minute, DBP—diastolic blood pressure, cDBP—central diastolic blood pressure, cPP—central pulse pressure, cSBP—central systolic blood pressure, cm—centimeter, kg—kilogram, ln—natural log transformation, m^2^—meter squared, m/s—meters per second, mmHg—millimeter of mercury, MVPA—moderate-to-vigorous physical activity, *n*—number, PWV—pulse wave velocity, RMSSD—root of mean successive differences, SBP—systolic blood pressure, SD—standard deviation, SDNN—standard deviation of normal R-R, * indicates *n* = 25.

## Data Availability

The datasets used and/or analyzed during the current study are available from the corresponding author on reasonable request.

## Supplementary Materials

The following supporting information can be downloaded at https://www.mdpi.com/article/10.3390/jcdd11100307/s1, Table S1: Cardiovascular and autonomic responses to prolonged sitting with and without prior exercise (*n* = 25).
