# Supplementary material for: Effect of Prior Moderate Aerobic Exercise to Prolonged Sitting on Peripheral and Central Cardiovascular Measures in Young Women
Source: J Cardiovasc Dev Dis. 2024 Oct 3;11(10):307. doi: 10.3390/jcdd11100307 (PMC11508224; doi:10.3390/jcdd11100307)
Supplement: Supplementary file 1 [file jcdd-11-00307-s001.zip › jcdd-3213328-supplementary.pdf]

Table S1. Cardiovascular and autonomic responses to prolonged sitting with and without prior exercise (n=25).

Outcome	Condition	1-hour of sitting (mean±SD)	2-hour of sitting (mean±SD)	3-hour of sitting (mean±SD)	Condition Effect *β* (SE) *(p-value)*	*d*
**SBP (mmHg)**	EX+SIT	109.66±1.68	108.58±1.78	111.67±1.69	.77 (1.18) *(.512)*	.08
	SIT	108.99±2.13	111.10±1.64	109.59±1.87		
**DBP (mmHg)**	EX+SIT	**68.74±1.42**	**68.14±1.80**	**67.69±1.57**	**2.18 (.91) (*.016*)**	**.29**
	SIT	**68.00±1.18**	**67.60±1.61**	**69.91±1.35**		
**MAP (mmHg)**	EX+SIT	87.40±1.37	86.80±1.45	87.85±1.13	1.53 (.79) (*.054*)	.19
	SIT	86.75±1.38	87.46±1.37	88.11±1.42		
**cSBP (mmHg)**	EX+SIT	99.94±1.80	97.64±1.79	98.88±1.33	1.39 (1.22) *(.255)*	.15
	SIT	97.81±1.92	99.29±1.98	99.26±1.78		
**cDBP (mmHg)**	EX+SIT	**71.18±1.28**	**69.88±1.72**	**69.48±1.53**	**1.99 (.93) *(.034)***	**.26**
	SIT	**69.73±1.28**	**69.92±1.74**	**72.09±1.36**		
**cPP (mmHg)**	EX+SIT	28.76±1.20	27.76±1.34	29.33±1.58	-.48 (1.00) (*.635*)	.08
	SIT	28.08±1.51	29.38±1.15	27.17±1.15		
**PWV (m/s)**	EX+SIT	4.72±.08	4.63±.08	4.72±.08	.02 (.06) *(.616)*	.05
	SIT	4.64±.09	4.73±.09	4.69±.10		
**AIX@75 (%)**	EX+SIT	26.52±1.82	24.96±1.51	24.75±1.37	-.99 (.87) (.253)	.11
	SIT	24.94±1.70	25.17±1.53	22.89±1.66		
**lnSDNN***	EX+SIT	3.65±.41	3.68±.40	3.76±.40	.04 (.04) *(.323)*	.13
	SIT	3.70±.33	3.82±.38	3.78±.38		
**lnRMSSD***	EX+SIT	3.31±.61	3.31±.59	3.52±.57	.01 (.06) *(.926)*	.02
	SIT	3.42±.46	3.51±.49	3.50±.52		

Abbreviations: ln; natural log transformation, m/s; meter per second, mmHg; millimeter of mercury, SD; standard deviation, SE; standard error, *d*; Cohen's d, Bold indicates significant change (p<0.05), * indicates n=26.
